# Supplementary material for: Storage Stability of Plant-Based Drinks Related to Proteolysis and Generation of Free Amino Acids
Source: Foods. 2024 Jan 23;13(3):367. doi: 10.3390/foods13030367 (PMC10855237; doi:10.3390/foods13030367)
Supplement: Supplementary file 1 [file foods-13-00367-s001.zip › foods-2789804-supplementary.pdf]

Table S1. Declared contents of PBDs, their name and manufacture company. The commercial names are in some cases translated to English.

Sample Name	Content	Commercial name	Company
Oat	Water, 16% oat, sunflower oil, inulin, sea salt	Oat	Naturli' Foods A/S
Oat/hemp	Water, 13% oat, ground hemp seeds (0.75%), salt	Oat and Hemp	Jörd, Arla Foods
Almond	Water, 2% almond, cane sugar, lithothamnium calcareum, sea salt, gellan gum, guar gum	Almond Drink	Naturli' Foods A/S
Roasted almond	Water, cane sugar, 2% roasted almond, lithothamnium calcareum, sea salt, gellan gum, guar gum	Roasted Almond	Naturli' Foods A/S
Pea	Water, 2.5% pea protein, agave syrup, rapeseed oil, dipotassium phosphate, calcium carbonate, calcium phosphate, oat oil, salt, vitamins (D2, B2, B12)	Pea	Dryk
Soy	Water, 10% soy beans	Soy Drink	Naturli' Foods A/S
Soy/rice	Water, 10% soy beans, rice, lithothamnium calcareum, vanilla aroma, sea salt	Soy with Calcium and vanilla	Naturli' Foods A/S

Table S2. Data for each of the stored PBDs on all 11 sampling days. Table showing pH, color measurement as applied in the CIELAB system, L*, black-white scale, where L* = 0 = black and L* = 100 = white; a*, red-green scale, with negative a* = green and positive a* = red; and b*, yellow-blue scale, with negative b* = blue and positive b* = yellow, % protein in whole sample and % protein in supernatant after centrifugation as a measure of protein solubility. For pH and color, the standard deviations were all under 3% and therefore not included for better readability. Protein solubility is tested ($p < 0.05$) by comparing protein content in day 0 before and after centrifugation and after centrifugation at day 364.

Sample	Day	pH	L*	a*	b*	Protein PBD (%)	Protein supernatant (%)
Oat	0	6.77	58.89	-0.33	10.02	0.456 ± 0.007[b]	0.164 ± 0.018[a]
	14	6.93	58.74	-0.21	10.22		
	28	6.69	58.76	-0.22	10.24		
	49	6.68	58.65	-0.21	10.14		
	91	6.88	54.50	0.04	9.89		
	133	6.46	54.12	0.12	9.97		
	175	6.45	54.41	0.20	9.93		
	217	6.77	54.30	0.31	10.07		
	259	6.74	54.15	0.37	9.68		
	301	6.52	53.68	0.18	9.81		
	364	6.54	53.97	0.44	9.92		0.226 ± 0.001[a]
Oat/hemp	0	6.80	54.42	-1.51	8.46	0.930 ± 0.003[b]	0.328 ± 0.000[a]
	14	6.82	54.50	-1.53	8.48		
	28	6.62	54.52	-1.27	8.18		
	49	6.57	53.60	-1.12	8.01		
	91	6.60	49.84	-0.88	7.71		
	133	6.12	49.58	-0.78	7.64		
	175	6.32	49.55	-0.67	7.60		
	217	6.53	49.36	-0.56	7.62		
	259	6.54	48.79	-0.47	7.48		

	301	6.37	48.60	-0.50	7.41		
	364	6.39	48.55	-0.40	7.47		0.334 ± 0.002[a]
Roasted Almond	0	7.60	55.84	0.48	7.87	0.441 ± 0.005[b]	0.265 ± 0.000[a]
	14	7.80	55.80	0.51	7.88		
	28	7.48	55.69	0.52	7.85		
	49	7.51	55.75	0.47	7.60		
	91	7.38	55.51	0.44	7.61		
	133	7.51	52.45	0.64	7.34		
	175	7.08	52.32	0.65	7.18		
	217	7.31	52.07	0.57	7.32		
	259	7.67	52.12	0.64	7.11		
	301	7.77	51.98	0.47	7.10		
	364	7.37	52.43	0.44	6.93		0.368 ± 0.002[ab]
Almond	0	8.08	59.32	-0.19	2.84	0.449 ± 0.019[b]	0.310 ± 0.003[a]
	14	7.74	59.41	-0.17	2.81		
	28	7.84	59.08	-0.17	2.65		
	49	7.69	59.20	-0.14	2.45		
	91	7.65	59.06	-0.20	2.41		
	133	7.74	55.87	-0.01	2.43		
	175	7.42	55.61	-0.01	2.26		
	217	7.46	55.78	-0.13	2.23		
	259	7.78	55.60	-0.11	2.34		
	301	7.87	55.38	-0.19	2.22		
	364	7.50	55.60	-0.19	2.25		0.312 ± 0.125[a]
Pea	0	7.62	63.75	-1.40	8.41	1.702 ± 0.035[c]	1.144 ± 0.003[a]
	14	7.66	63.52	-1.45	8.57		
	28	7.75	63.80	-1.39	8.62		
	49	7.67	64.02	-1.28	8.52		
	91	7.66	60.59	-1.24	8.22		
	133	7.77	59.94	-1.01	8.18		
	175	7.45	59.74	-1.02	8.28		
	217	7.63	59.61	-1.13	8.16		
	259	7.63	59.52	-1.00	8.22		
	301	7.75	59.70	-1.03	8.17		
	364	7.61	59.57	-1.03	8.01		1.173 ± 0.000[b]
Soy	0	7.18	62.48	-3.00	11.57	4.223 ± 0.182[b]	3.201 ± 0.033[a]
	14	7.31	62.78	-3.00	11.51		
	28	7.24	62.61	-2.95	11.40		
	49	7.19	62.47	-2.82	11.22		
	91	7.17	58.55	-2.47	10.60		
	133	6.77	58.51	-2.55	10.42		
	175	7.05	58.71	-2.49	10.27		
	217	7.26	58.69	-2.33	10.10		
	259	7.32	58.51	-1.94	8.52		
	301	7.17	58.48	-2.36	9.31		
	364	7.24	58.40	-2.16	9.42		3.222 ± 0.001[ab]
Soy/rice	0	7.94	58.70	-1.10	10.73	2.617 ± 0.584[a]	2.884 ± 0.065[a]
	14	7.95	57.81	-0.82	10.53		
	28	7.87	58.24	-0.70	10.53		

	49	7.88	57.73	-0.40	10.33	
	91	7.88	53.55	-0.02	9.81	
	133	7.34	53.32	0.02	9.86	
	175	7.56	53.37	0.16	9.80	
	217	7.75	53.26	0.34	9.69	
	259	7.76	52.71	0.54	9.62	
	301	7.47	52.80	0.32	9.68	
	364	7.54	52.85	0.52	9.68	2.984 ± 0.000[a]

Table S3. Contents of free AAs in PBD at days 0, 175 and 364 in mg AA/g protein, n=3. Letters indicate significant difference ($p \leq 0.05$) in contents of each free AAs in a PBD over the storage period. Not detected (ND).

Free AA	Day	Oat	Oat/hemp	Almond	Roasted almond	Pea	Soy	Soy/rice
β-Alanine	0	0.22 ± 0.03[ab]	0.07 ± 0.00	0.04 ± 0.01	0.08 ± 0.02	0.03 ± 0.00	0.05 ± 0.00	0.10 ± 0.00
	175	0.19 ± 0.02[a]	0.07 ± 0.00	0.05 ± 0.00	0.07 ± 0.01	0.03 ± 0.00	0.06 ± 0.01	0.11 ± 0.01
	364	0.24 ± 0.04[b]	0.08 ± 0.01	0.04 ± 0.00	0.09 ± 0.00	0.04 ± 0.00	0.04 ± 0.00	0.10 ± 0.01
Ethanolamine	0	0.11 ± 0.00[a]	0.17 ± 0.02[a]	0.17 ± 0.02	0.14 ± 0.02	0.02 ± 0.01	0.04 ± 0.00	0.06 ± 0.00
	175	0.13 ± 0.02[a]	0.20 ± 0.01[a]	0.21 ± 0.04	0.14 ± 0.01	0.02 ± 0.00	0.05 ± 0.01	0.06 ± 0.00
	364	0.25 ± 0.06[b]	0.32 ± 0.03[b]	0.17 ± 0.03	0.17 ± 0.01	0.02 ± 0.00	0.03 ± 0.00	0.06 ± 0.00
γ-Aminobutyric acid	0	0.61 ± 0.01	0.30 ± 0.02	0.57 ± 0.06	0.46 ± 0.04	0.03 ± 0.00	0.05 ± 0.01	0.12 ± 0.00
	175	0.79 ± 0.14	0.32 ± 0.02	0.56 ± 0.10	0.43 ± 0.02	0.05 ± 0.01	0.06 ± 0.01	0.13 ± 0.01
	364	0.65 ± 0.03	0.40 ± 0.03	0.53 ± 0.05	0.53 ± 0.04	0.05 ± 0.00	0.06 ± 0.00	0.12 ± 0.01
Orthinine	0	0.06 ± 0.00[a]	0.08 ± 0.01	0.02 ± 0.00	0.02 ± 0.00	0.01 ± 0.00	0.01 ± 0.00	0.01 ± 0.00
	175	0.07 ± 0.01[a]	0.10 ± 0.02	0.02 ± 0.01	0.02 ± 0.00	0.03 ± 0.00	0.01 ± 0.00	0.01 ± 0.00
	364	0.16 ± 0.01[b]	0.11 ± 0.01	0.02 ± 0.00	0.02 ± 0.00	0.03 ± 0.00	0.00 ± 0.00	0.02 ± 0.00
Aminobutyric acid	0	ND	ND	ND	ND	ND	ND	ND
	175					0.01 ± 0.00[a]		
	364					0.03 ± 0.00[b]		
Aminoisobutyric acid	0	ND	ND	ND	ND	ND	ND	0.08 ± 0.00[b]
	175							0.09 ± 0.00[c]
	364							0.00 ± 0.00[a]
Anserine	0	ND	ND	ND	ND	ND	ND	0.02 ± 0.03
	175						ND	0.01 ± 0.01
	364						0.01 ± 0.00	0.02 ± 0.01
Carnosine	0	ND	ND	ND	ND	ND	ND	0.04 ± 0.00
	175						ND	0.04 ± 0.00
	364						0.02 ± 0.00	0.04 ± 0.00
Citrulline	0	0.07 ± 0.01	ND	ND	ND	ND	ND	0.01 ± 0.00
	175	0.05 ± 0.01						0.01 ± 0.00
	364	ND						0.01 ±0.00
Hydroxyproline	0	0.07 ± 0.00[b]	0.01 ± 0.00	ND	ND	ND	ND	ND
	175	0.07 ± 0.01[b]	0.01 ± 0.00					
	364	0.04 ± 0.00[a]	0.01 ± 0.00					
Taurine	0	0.04 ± 0.01[b]	ND	ND	ND	ND	ND	ND
	175	0.02 ± 0.00[a]						
	364	ND						
